# Editorial Chit-Chat

**Published:** 1879-04

**Authors:** 


### Editorial Chit-Chat.

---

THE CREMATION SOCIETY has another member in the person of Jacob Swartz, Esq. He joined at Max Haight's editorial supper.

---

DELINQUENTS.—We are weeding them from our books as rapidly as possible. Send in your subscriptions and save being dropped. It is a shame to be in debt to so good a journal as this.

---

POSTAGE STAMPS—Do not send us any postage stamps above the denomination of three cents. We will always accept three, two and one cent postage stamps in payment for subscription, but none of higher denomination.

---

THE SPRING FLOWERS.—And now search for trailing arbutus. We hear of blossoms that have already been found upon the hillsides. The plucky little crocus has long since peeped through the snow, and given us promise of an early spring.

---

JOHN D. WILLIAMS.—This gentleman has served the city faithfully and well as one of its Supervisors. No man in the city has a better knowledge of the county's business affairs, and is better qualified to be entrusted in executing such business. It is greatly to the discredit and disadvantage of his ward that he was not re-elected.

---

DR. H. E. REINHOLD, a popular and successful physician of Williamsport, Pa., died in that city, March 6th.

Dr. Reinhold had a large practice, and was more widely known than any physician in his neighborhood. He was a skillful practitioner, a lovable companion, and a christian gentleman. He is a great loss to the community in which he dwelt.

---

THE DIME CONCERTS.—During the winter, Park Church Choir has been giving the people a series of delightful concerts. The church has been crowded at every one of them. Mrs. Gibson's sweet voice is heard to the best advantage as a concert singer. The entire quartette is individually good, and have sung to full houses in neighboring towns.

We will forgive them for that jubilee business, in their last concert, if they will do so no more.

THE JULY NUMBER—our friends will remember, will not be issued until the 15th of the month, by reason of the Editor's vacation, which occurs in the month of June.

---

THE CHINESE BILL.—"The National Workingmen's Assembly of San Francisco," in their resolutions to the President, urging him to sign the bill, make use of the following threat: "Is the only means that will prevent a terrible calamity and utter annihilation of the Chinese of the Paciffc coast, which is sure to follow the veto of the bill!" And how these same fellows howled and raved over the massacre of the missionaries in China, a few years ago. We will warrant that a majority of the members of this same workingmen's assembly are foreigners themselves, with no better right in this country than the Chinese and without the merit of being half as useful and law-abiding.

---

THAT STORY OF FRITZ, in the last BISTOURY, seemed to find a responsive throb of recognition, if not approval, in many a Journalist's heart, if we may judge from the extensive copying it received throughout the country.

The working of a sound, hearty boy's mind is worth the watching as it finds expression in his sayings and doings. There is a world of humor and wisdom in a robust lively boy. Keep track of him (if you can) for three months, and wonder from what mysterious fountain flows the mirth, mischief and philosophy of a five-year-old.

---

LIFE-SAVING APPARATUS.—We read of an unusual number of wrecks with large loss of life, this winter. In many instances, the life-saving stations along the coast were unable to render assistance, by reason of their defective appliances for that purpose. In severe storms, when the wrecked vessel is being tossed about upon the huge waves, it is often impossible for the shore-men to throw a line over her from their mortar. Several failures of this sort are mentioned. Why should not a mortar, chain, and line be carried upon every vessel? It would be much easier to fire a ball ashore, than for the shoremen to shoot a ball over the vessel. It has always seemed to us that this apparatus should be carried aboard every vessel, for it is certainly easier to hit a continent with a cannon ball, than a bobbing, sinking ship.

CITY ARCHITECTURE.—Since the advent of Mr. W. H. Hayes, the architect, among us, our city begins to exhibit evidences of his masterly skill. Before, our dwellings and business edifices all seemed to be fashioned after one design, giving them a very ordinary appearance. But now, Mr. Hayes has lifted us from the rut and given us lessons in architectural beauty until our city blossoms with the results of his genius. Mr. Hayes has been of real value to us, and we are glad to note his unbounded success.

---

A QUEER DREAM.—A lady falteringly, and with tears in her eyes, related to us that she dreamed an angel came to her bedside and whispered in her ear three times, with startling distinctness, "your child will die, your child will die, your child will die!" She was much alarmed, inasmuch as her child was ill, and anxiously inquired whether the little one's symptoms were really worse. We tried to quiet her fears, and, notwithstanding the child was not very sick, it sank into a sound sleep that very night, and when the mother awoke in the morning, she found the dear little one——wide awake, and well as ever!

---

THE DISTRICT TELEGRAPH.—It is a great convenience, but when you ask one of your servants to ring for a messenger, you must be sure that he knows how.

Through the telephone, we directed our man at the Institute to ring for a messenger, and that when he arrived, to send him to our dwelling. He rang; presently he shouted to us through the telephone: "What are all these fire engines here for? They are getting ready to squirt on the house, better come over, quick!"

We went over to find that he had made the fire alarm, not knowing, he said, "how the blamed thing worked."

---

A BEAUTIFUL PLACE is Maxey Haight's new hotel. A paragon of neatness and exquisite taste in all its appointments. The frescos upon the ceilings are works of art, especially is this the case in the cozy little room assigned to the ladies for a restaurant. The billiard hall, with all its paraphanalia, is elegant, tasteful; a more delightful resort for gentlemen cannot be found. The hotel is intended as a stopping place for those gentlemen known as "commercial travelers," and Maxey has supplied everything needful for their comfort and pleasure. The sleeping apartments are especially attractive, and that the place will be patronized by the gentlemen for whom they have been provided, cannot be doubted. Maxey Haight's hotel is one of the institutions of the city to be shown to strangers;—it is a beautiful place to visit.

---

OUR CONTRIBUTORS.—We need not call the attention of our readers to the excellence of our contributions. The same corps of writers have been retained from the very beginning of the magazine, no periodical being more largely quoted from than the BISTOURY. It is gratifying to receive assurances, from all over the country, in very many complimentary letters, that the BISTOURY is so thoroughly appreciated. It is encouraging to receive such missives, and assure our friends that we appreciate them even if wedo not print them.

---

THE PARK.—*The Daily Advertiser* advocates the purchase, by the city, of Eldridge Park. In this, the *Advertiser* is right. There is no one thing that has given Elmira so much notoriety as Eldridge Park. If the city is remembered by a stranger for nothing else, the beautiful Park is always spoken of. During the years that Dr. Eldridge's generosity kept it open to the public, as many people were attracted to it, visiting its delightful flowers, statuary and drives, as came to the more pretentious State Fair. The County thought it worth the while to invest thousands of dollars in the purchase of State Fair Grounds, with a view of inducing strangers to our borders; why cannot the city, with equal propriety, expend five or six thousand dollars yearly, to secure a Park that will not only attract thousands of people to our city, but also prove a constant delight as a resort for her own citizens ?

The only argument perhaps, that will be offered against the acquirement of this beautiful Park is that, in view of the stringent times, the people can illy afford increased taxation. True enough; but should we wait for better times, the opportunity for making so desirable and advantagious a purchase will have escaped us. The Park, as it now stands, is finished and complete. Three times the price asked for it has been expended in its construction. It is known and praised throughout the land. For years,

Elmira's Park has been her chief attraction to strangers. It seems a pity now to lose it, particularly when it can be secured for a trifle that will never be felt by the individual tax-payer. We vote for the purchase of Eldridge Park!

---

COL. LUTHER CALDWELL.—This lecturer has done heroic work throughout the country in the cause of temperance. He is a gentleman of most pleasing mein, winning friends wherever he goes. Unlike many of the reformed bummers who have entered the lecture field solely as a means of replenishing their exhausted exchequers, and who offend society with recitals of the experiences of their wretched, beastly lives, Col. Caldwell appeals to the finer feelings of his audiences, convincing with his logic and eloquence. His appeals to the people are masterly, having won for him a fame only excelled by Mr. Murphy, to whom he is superior as an orator and earnest worker.

---

THE CEMETERIES AND DIPHTHERIA—APPARENT CONFIRMATION OF DR. UP DE GRAFF'S THEORY :—

The theory which Dr. Up de Graff has advanced, as affording a possible clue to the source of diphtheria in this city, has brought to our mind facts which appear to fortify the position taken by him. We state these facts briefly: In the summer of 1877, portions of the town of Hornellsville, were scourged with diphthera. The disease was most virulent and fatal in, and in fact was almost confined to, districts whose wells were supplied by natural water courses arising in Mount Hope, the village cemetery of Hornellsville. Residents from other parts of the town were comparatively free from the visitation of the disease.

During the summer and winter of 1877, and last summer the diphtheria prevailed in the town of Watkins, with such fatal severity, that whole families of children were swept away, and the town almost plunged into a panic before its direful progress. Now it is stated that the disease in Watkins, committed its ravages in those portions only whose drinking water is supplied from courses having their rise on the hill west of the town. On this hill is, and has been for years, located the village cemetery, the Lake View.

In both towns those who lived away from these natural water courses, on higher ground, or at more remote distances, escaped the fury of the scourge. These facts certainly seem to support the Doctor's theory. They are at least suggestive, and carry a significance which our health authorities should not ignore.—*Daily Free Press.*

Our readers will find other confirmations of the theory, by referring to Our Correspondence. That wells located near cemeteries are liable to become contaminated with drainings from the decomposing bodies, seems too evident to call for a warning against the danger. We are glad the BISTOURY has awakened some interest in the matter, and that people may thereby be induced to abandon all wells located in unfavorable situations.

---

A FLORIST'S DEATH.—He was a modest, unassuming man, known to but few, and those only, who, like himself, were lovers of the beautiful flowers. Flowers were his pets—his inspiration. A man of but slender advantages, he could not be called cultured, save where his wonderful knowledge of flowers rendered him eloquent when speaking of them. He knew the name and nature of every plant seemingly, upon the habitable globe, and his mild blue eye would sparkle, revealing a passionate love for the theme, as he modestly told of the beauty of some new acquisition to his green-house. Seldom was he seen upon the streets; no companions had he, save the flowers, among which he worked the live-long day, receiving from them lessons in fragrance, beauty and contentedness of character rarely to be found. We loved the old man, and we both loved the flowers among which he toiled, cementing between us a friendship lasting through many years. Few there be who knew the kind, patient old florist, or who will miss him, now that he is dead. Our acquaintance with him was a delightful one, for a gentler, lovelier spirit than Hugh M. Moore, was never revealed to us.

---

TO THE PROFESSION.—We have over five thousand physicians on our subscription list. We are sure that this number could be greatly increased if medical men had an opportunity of examining the BISTOURY. We therefore mail sample copies to many physicians throughout the country, and ask them to examine our "Medical Items and News" department. This feature of our journal is one that appeals especially to the busy practitioner of medicine, and cannot fail to be of great advantage to him. We use much care in editing these items, giving in condensed form, all that is transpiring in the medical world, and culling our information from more than fifty of the best medical journals published.

We ask at the hands of the medical man, a careful examination of this work, when we are sure to secure him as a permanent subscriber. Every three years we publish a full index to the Medical Department, arranging it carefully by the names (common and technical) of diseases treated upon. It makes a book of 330 pages, in which is a complete epitome of what has transpired in medicine during that time.

When it is remembered that all this is furnished for the small sum of fifty cents a year, it seems as though every physician in the land would become a subscriber.

Please examine the current number, and send us your subscription.

THE GREAT ENGLISH PHYSICIAN :—

We have refrained from noticing a card published by T. E. Doolittle, Esq., concerning his course as counsel for the pretender Burner, in New Haven, although there were imputations in it as unjust as were some of his statements in court. The *Palladium* yesterday had some vigorous comments upon his treatment of the eminent scientists who testified—treatment which was certainly inexcusable on any grounds. Concerning some other points we have preferred to believe that Mr. Doolittle was deceived by the falsehoods of his client, as others have been. Certainly no attorney of the standing of Mr. Doolittle would have deliberately said in court that the author of the *Bistoury* articles exposing Burner was "sent to jail" for publishing them. Burner must have so informed his counsel. The statement is wholly false. Mr. Doolittle must have been deceived. He could not have made the statement on his own responsibility, and then have published a card in the *Palladium* suggesting that his "adversaries" would under *any* circumstances be guilty of "fabricating testimony,"—*Hartford Courant.*

At last, this impudent traveling mountebank has met his just punishment. When the BISTOURY first took him in hand, two years ago, exposing his fraudulent practices, and showing what his qualifications were for practicing medicine, he made a great bluster, filling the newspapers with threats of the terrible fate he meant to subject the BISTOURY to. But the BISTOURY kept right along, showing that the man Burner, was an auctioneer from Dayton, Ohio, that he never read medicine, never attended any college, never was in England, nor scarcely from his own county, before he came to be a traveling "Great English Physician." Letters were published from reliable business men from Dayton, setting forth the facts in the case, and one from Dr. Gleason, of Philadelphia, showing that the man Burner, was using Dr. Gleason's lectures and book, claiming them as his own.

When Burner learned that the BISTOURY had posession of this knowledge, and meant to print it, he left—left so suddenly that no one knew precisely where he went. But the BISTOURY learned his where-a-bouts and mailed one thousand copies of the journal, to his new field of operations. Again he was driven away to "pastures new," to which the journal again followed him, until at last, he left the state entirely, and went to Connecticut. There, Mr. Hubbard of the *Courant*, took charge of the swindler, (for almost destroying the life of his brother, with his "tape worm dodge"); and, after prosecuting him for malpractice, (for which he was forced to pay several thousand dollars in settlement), arrested him for swindling, breaking up his "doctor business" entirely. Where the "Great English Physician" now is, cannot be learned. Perhaps gone to join Leon Lewis, of the *Mystery*. But of one thing the good people of the States of New York and Connecticut can be assured; namely, the "Great English Physician" (from Newark, Ohio,) with his liveried valet, and Dr. Gleason's lectures will not reappear in their midst, to gull a credulous community.

---

FRITZ VISITS THE CONSERVATORY.—Fritz, forbidden to play in the rain, wandered listlessly, almost aimlessly, into the conservatory. He was alone, or thought he was, for his father, who occupied an elevated position behind a climbing rose—looking for red spiders—was unperceived by him as he silently watched the boy's manœuvers. Fritz leaned against the cutting bench, in a languid, dispirited sort of manner, and, thrusting both elbows into the sand, rested his chin upon his hands and lazily floundered his feet about until they came in contact with a stack of flower-pots that fell with a crash upon the hard lattice. At this, he quickly turned, eyed the ruins for a minute, simply exclaiming, without the slightest alarm or concern,

"Gracious, they're busted all to flinders!"

Stepping over and among the fragments, he broke a long branch from a flowering begonia with which he stirred up the gold fish until two or three sought refuge outside the vase, where they lay flopping upon the floor. He watched their flip-flaps with evident satisfaction, renewing their exertions with the toe of his boot whenever their efforts flagged. Presently the canary, suspended overhead in a cage, commenced his morning bath, sprinkling water upon the boy. Fritz stood back and watched him for a moment, and then addressed the bird as follows:

"Say, old feller! I know a better way'n that! Jest let me show you how to take the bulliest kind of a bath—jest hold on a minute!"

He picked up the nozzle of the hose, turned on the water and directed the full stream upon the defenseless bird. The first shot knocked him from the perch, while the inundation that followed nearly drowned the astonished little creature. Just then, a bevy of pigeons alighted upon the glass roof, overhead, seeking a warm place for their feet. Fritz dropped the hose and watched—gazed intently upon them while they strutted and cooed over the transparent roof, at which he manifested as much delight as the birds themselves. Suddenly his face brightened—his black eyes sparkled—his fingers moved nervously, betokening the speedy termination of the pigeon frolic, likely, too, in some disastrous manner. He looked about—saw the long-handled pruning shears—turned the handle toward the glass immediately beneath a strutting pigeon—moved it up and down while he took aim along its length, and, as the final lurch was about to be made, the father shouted, when Fritz dropped the pole and looked aloft much mystified and surprised.

"What are you up to now, my boy?"

"O, nothin', pa, I was jes only thinkin' how jolly nice 'twould be to *boost* that pigeon!"

